# Data of heavy metals biosorption onto *Sargassum oligocystum* collected from the northern coast of Persian Gulf

**DOI:** 10.1016/j.dib.2016.05.035

**Published:** 2016-05-24

**Authors:** Sedigheh Delshab, Esmaeil Kouhgardi, Bahman Ramavandi

**Affiliations:** a Environmental Department, Bushehr Branch, Islamic Azad University, Bushehr, Iran; b Environmental Health Engineering Department, Faculty of Health, Bushehr University of Medical Sciences, Bushehr, Iran; c Systems Environmental Health, Oil, Gas and Energy Research Center, Bushehr University of Medical Sciences, Bushehr, Iran

**Keywords:** Biosorption, Hg^2+^ ion, Cd^2+^ ion, Cu^2+^ ion, *Sargassum oligocystum*

## Abstract

This data article presents a simple method for providing a biosorbent from *Sargassum oligocystum* harvested from the northern coast of Persian Gulf, Bushehr, Iran. The characterization data of *Sargassum oligocystum* biochar (SOB) were analyzed using various instrumental techniques (FTIR and XPS). The kinetics, isotherms, and thermodynamics data of Hg^2+^, Cd^2+^, and Cu^2+^ ions onto SOB were presented. The maximum biosorption capacity of SOB to uptake Hg^2+^, Cd^2+^, and Cu^2+^ ions from aqueous solution was obtained 60.25, 153.85, and 45.25 mg/g, respectively. The experimental data showed that biochar prepared from *Sargassum oligocystum* is an efficient and promising biosorbent for the treatment of heavy metals-bearing wastewaters.

**Specifications Table**

**Table:** 

Subject area	*Environmental Engineering*
More specific subject area	*Biosorption*
Type of data	Table, figure
How data was acquired	-The uptake of metals by the biosorbent (*q_e_*) was determined based on the difference between the initial and final concentration of metals.- Fourier transform infrared (FTIR) spectroscopy (Shimadzu 4300), X-ray photoelectron spectrometer (KRATOS AXIS 165), and atomic absorption spectroscopy (AAnalyst 200 Perkin-Elmer).
Data format	Analyzed
Experimental factors	– *Sargassum oligocystum* biochar (SOB): The SOB was provided from brown alga of *S*. *oligocystum* at 350 °C. – Data of SOB were collected for Cd^2+^, Cu^2+^, and Hg^2+^ removal from solution. – - The data related to kinetics, isotherms, and thermodynamic was presented.
Experimental features	*S*. *oligocystum* biochar as Cd^2+^, Cu^2+^, and Hg^2+^ biosorbent.
Data source location	Bushehr University of Medical Sciences, Bushehr, Iran, GPS: 28.9667°N, 50.8333°E.
Data accessibility	Data are available with the article.

## Value of the data

• A biochar provided from *Sargassum oligocystum* was applied for attenuating Cd^2+^, Cu^2+^, and Hg^2+^ from aqueous solution.
• Information of this data article including, isotherm, kinetic, and thermodynamic parameters will be informative for modeling and predicting the biosorption capacity and mechanism of heavy metal removal by algae.
• This data set will be beneficial for the scientific community wanting to scale up and design a biosorption column with *S*. *oligocystum* biochar as medium for the removal of heavy metal- bearing waters or wastewaters.

## 1 Data

**Fig. 1:**
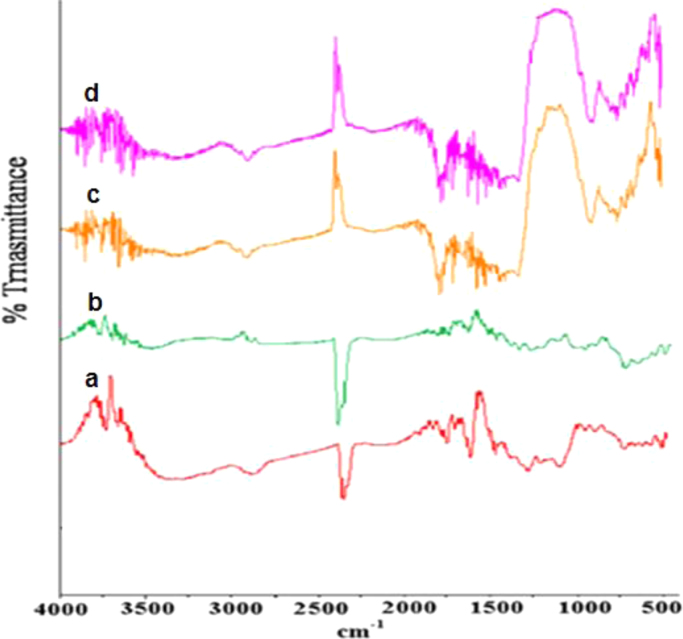
FTIR spectra for (a) fresh SOB, (b) Cd- loaded, (c) Cu- loaded SOB, and (d) Hg- loaded SOB.

**Fig. 2:**
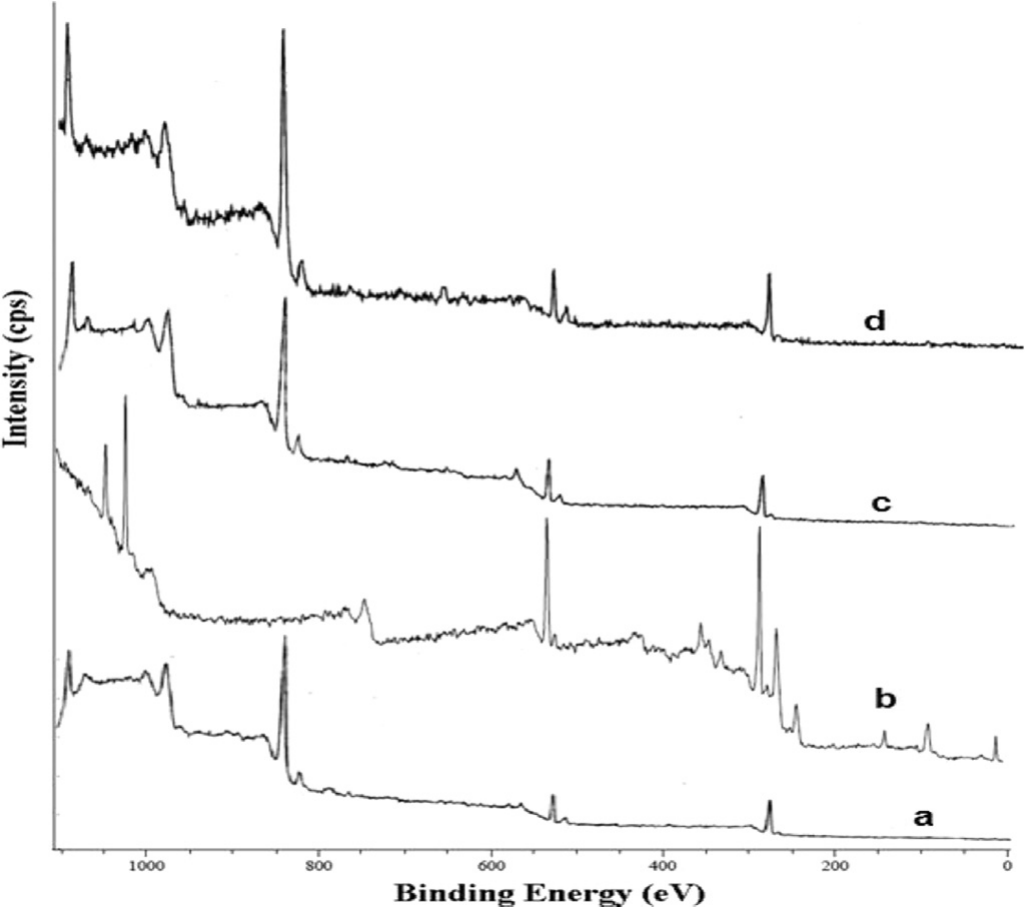
XPS wide scan spectra for (a) fresh SOB, (b) Cd- loaded SOB, (c) Cu- loaded SOB, and (d) Hg loaded SOB.

**Fig. 3:**
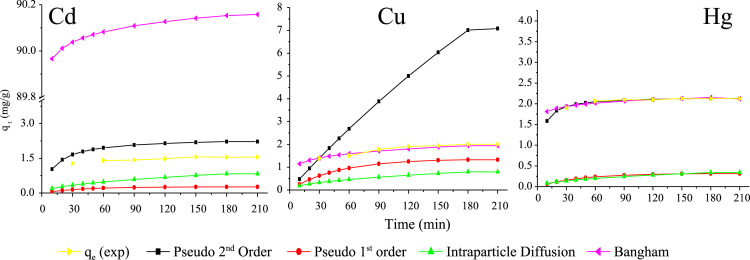
Biosorption kinetics for Cd, Cu, and Hg using SOB (120 rpm, 24 °C, 10 g/L biosorbent, optimum pH).

**Fig. 4:**
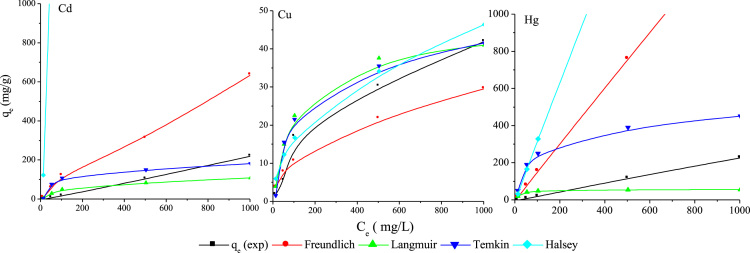
Biosorption isotherm for Cd, Cu and Hg using SOB (120 rpm, 24 °C, 10 g/L biosorbent, 2 h, optimum pH).

The FTIR of the fresh and used SOB particles at wave numbers from 500 to 4000 cm^−1^ are shown in Fig. 1. The X-ray photoelectron spectroscopy (XPS) of fresh SOB and Cd^2+^, Cu^2+^, and Hg^2+^ loaded SOB is also depicted in Fig. 2. Data of this article including, kinetics, isotherms, and thermodynamic analysis was calculated using models provided in Table 1. The data of kinetics and isotherms for biosorption of heavy metals (cadmium, copper, and mercury ions) onto SOB were first depicted in Fig. 3, Fig. 4. Through Fig. 3, Fig. 4 and Table 1, the kinetics, isotherms, and thermodynamic parameters were calculated and summarized in Table 2, Table 3.

## 2 Experimental design, materials and methods

### 2.1 Materials

The mass of brown algae (*S*. *oligocystum*) was harvested from the Persian Gulf, Bushehr coast, Iran. The collected *S*. *oligocystum* masses were first washed with seawater for removing debris and sand and then shipped to the laboratory. In the laboratory the biomasses of *S*. *oligocystum* was washed extensively with running tap water for around 30 min followed by deionized water to remove impurities. After that, the biomass was dried at 350 °C for 2 h in a Muffle Furnace. The dried biomass (biochar) was ground to achieved a particle size of a 200-mesh (*Φ*= 0.074 mm). The obtained particles were used in the experiments as *S*. *oligocystum* biochar (SOB).

### 2.2 Experimental design

Biosorption batch tests with the prepared SOB were conducted in 100 mL flask and stirred at 120 rpm in a shaker–incubator instrument (Parsazma Co., Iran). Each test contained of preparing 50 mL of adsorbate (Cd, Cu, and Hg) with a given initial concentration. The initial pH of the solution was regulated as required by addition of 0.1 M HCl and NaOH solutions. After the sample reached equilibrium, the sample was passed through a 0.42 µm- filter, and the final concentration of the contaminant was determined. The amounts of contaminant adsorbed per gram of SOB, *q_e_* (mg/g), were obtained as follows [3], [4]:

(1) $$q_{e}=\frac{C_{0}-C_{e}}{M}$$

where *C*_0_ and *C_e_* (mg/L) are contaminant concentrations at initial and equilibrium, respectively. *M* (g/L) denote the dry mass of SOB in the solution.

Isotherms analyses were performed with various initial concentrations of Cd^2+^, Cu^2+^, and Hg^2+^ (see *x*- axis of Fig. 4), contact time of 8 h, solution temperature 24 °C, and mixing intensity of 120 rpm. Kinetic tests were done using a known initial concentration at 24 °C for a determined contact time (*t*=0–210 min).

The thermodynamics of biosorption process of Cd^2+^, Cu^2+^, and Hg^2+^ onto SOB was assessed using a 100-mL flask, containing 50 mL of pre-determined concentration of the adsorbate, 10 g/L SOB, mixing intensity of 120 rpm. This test was performed at designated temperature (24 °C). The thermodynamics of contaminants biosorption onto SOB was analyzed using an estimated change in biosorption free energy (Δ*G*°), biosorption enthalpy (Δ*H*°), and biosorption entropy (Δ*S*°) as defined in the Table 1.

All biosorption tests were performed at least in duplicate to ensure the reproducibility of data, and the average values are stated herein. Blank tests containing no SOB were also undertaken.

### 2.3 Analytical methods

FTIR spectra for fresh and used SOB samples were recorded by the KBr pellets method operated on FTIR spectrophotometer (Shimadzu 4300, Japan). Data processing was performed to transform absorbance into transmittance percentage showing wavelength peaks. The residual concentration of Cd^2+^, Cu^2+^, and Hg^2+^ ions in the treated solutions was analyzed using an atomic absorption spectroscopy (AAnalyst 200 Perkin-Elmer). The initial and final pH of the solution was measured using a pH meter (METLER TOLEDO FE20). The surface of the SOB samples before and after heavy metals adsorption was analyzed by using an X-ray photoelectron spectrometer (XPS, KRATOS AXIS 165). The XPS was operated at a base pressure of 8×10^−8^ Pa and pass energy of 23.5 eV. The calibration of the spectra was done by graphitic carbon as the energy referenced to C1s at 284.6 eV.

The value of correlation coefficients (*R*^2^) and the standard deviation (*SD*) of data was used to assess the goodness of the kinetic and isotherm models. *SD* can be expressed as:

(2) $$\mathrm{SD}=\sqrt{\frac{1}{n}\sum_{i=1}^{n}{\left(X_{i}-\overset{}{\overset{¯}{X}}\right)}^{2}}$$

where *X*_1_, *X*_2_, …, *X_n_* are the obtained values of the measurements, $\overset{}{\overset{¯}{X}}$ is the average value of the measurements, and *n* is the size of the sample.

## Figures and Tables

**Table 1:** Isotherm and kinetic models and thermodynamic equations used in this data article [1], [2].

Model	Functional form	Plotting
Langmuir	$\frac{q_{e}}{q_{m}}=\frac{K_{L}C_{e}}{1+K_{L}C_{e}}$	$\frac{1}{q_{e}}\mathrm{vs}\frac{1}{C_{e}}$
Freundlich	$q_{e}=K_{f}C_{e}^{1/n}$	log $m_{1}=f_{2}/f_{1}$
Temkin	$\frac{q_{e}}{q_{m}}=\frac{\mathrm{RT}}{b_{T}}\ln\left(k_{T}C_{e}\right)$	$q_{e}\;\;\;\mathrm{vs}\;\ln C_{e}$
Halsey	$q_{e}=k_{H}/{CH}_{e}^{1/n_{H}}$	log $q_{e}\;\mathrm{vs}\;l\mathrm{og}C_{e}$
Pseudo 1st order	$\frac{\mathrm{dq}}{\mathrm{dt}}=k_{1}(q_{e}-q_{t})$	log ${(q}_{e}-q_{t})vs\;t$
Pseudo 2nd order	$q_{t}=\frac{q_{e}^{2}k_{2}t}{1+q_{e}k_{2}t}$	$\frac{t}{q_{t}}vs\;t$
Intraparticle diffusion	$q_{t}=k_{\mathrm{ip}}t^{0.5}$	$q_{t}\mathrm{vs}\;\;\;\;t^{0.5}$
Bangham	$\ln\left(1-\frac{q_{t}}{q_{e}}\right)={-k}_{B}t$	$\ln\left(1-\frac{q_{t}}{q_{e}}\right)vs\;t$
Thermodynamics	*ΔG*°= −*RT* ln *K_Th_*; ln *K_Th_*= (*ΔS*°/*R*)−(*ΔH*°/*RT*); *ΔG*°= *ΔH*°− *TΔS*°	ln *K_T__h_* vs 1/*T*

*q*_*m*_ = maximum adsorption capacity, *k*_*L*_= Langmuir constant, *k*_*f*_ and *n* = Freundlich constants; and *k*_*T*_ and *b*_*T*_ = Temkin constants, *k*_1_ = rate constant of pseudo-first order model, *k*_2_ = rate constant of pseudo-second order model, *k_ip_* = intraparticle diffusion constant, *k_B_*= Bangham constant, *q*_*t*_= adsorbed amount at any time, *q*_*e*_ = adsorbed amount at equilibrium, *R* = universal gas constant, *T* = absolute temperature in Kelvin (298 K), *ΔG°*= Gibbs free energy change (kJ/mol), *Δ*H*°*= enthalpy change (kJ/mol), *ΔS°*= entropy change (kJ/mol K), and *K*_*Th*_= thermodynamic equilibrium constant (mL/g).

**Table 2:** Kinetics parameters for Cd, Cu and Hg adsorbed onto SOB.

Parameter	Cd	Cu	Hg
*q*_*e*, exp_ (mg/g)	2.385	2.004	2.126
Pseudo 1st order			
*q*_*e*,cal_ (mg/g)	0.610	1.362	0.318
*k*_1_ (min^−^^1^)	−0.041	0.020	0.022
*R*^2^	0.901	0.984	0.916
*SD*	0.069	0.089	0.203
Pseudo 2nd order			
*q*_*e*,cal_ (mg/g)	1.424	2.101	2.173
*k*_2_ (g/mg.min)	0.702	0.029	0.124
*R*^2^	1.000	0.946	0.999
*SD*	0.032	0.028	0.019
Intraparticle diffusion			
*K_ip_* (mg/g.min^0.5^)	0.025	0.120	0.025
*R*^2^	0.879	0.999	0.879
*SD*	0.045	0.0002	0.045
Bangham			
*K_BM_*	0.126	−142.032	−124.362
*α*	5.99E−04	0.182	0.059
*R*^2^	0.955	0.937	0.926
*SD*	4.20E−05	0.022	0.008

**Table 3:** Isotherms and thermodynamic parameters for Cd, Cu, and Hg adsorbed onto SOB.

Parameter		Cd		Cu			Hg
*q*_*e*,exp_ (mg/g)		217.155		42.111			229.993
Freundlich							
*K_f_* (L/g)		1.128		1.414			1.787
*n*		0.983		2.267			1.026
*R*^2^		0.994		0.875			0.977
*SD*		0.044		0.024			0.019
Langmuir							
*K_L_* (L/mmol)		0.005		0.011			0.066
*q_m_* (mg/g)		153.85		45.250			60.250
*R*^2^		0.998		0.984			0.749
*SD*		0.025		2.330			0.099
Temkin							
*K_T_* (L/mmol)		0.115		0.122			0.187
*R*^2^		0.939		0.955			0.993
*SD*		0.069		5.746			0.095
Halsey							
*k_H_* (L/g)		0.359		0.165			0.253
*n_H_*		−0.687		−2.267			−1.026
*R*^2^		0.997		0.874			0.976
*SD*		0.029		0.523			0.028
Thermodynamic parameters							
at 297K	*∆G*° (KJ/mol)		*ΔS*° (KJ/mol)		*∆H*° (KJ/mol)	*R*^2^/ *SD*	

Cd	−2.451		0.0019		−1.852	0.986/ 0.007	
Cu	−0.632		0.829		−0.381	0.914/0.006	
Hg	−16.157		0.022		−9.395	0.992/0.026	
